# Perspective taking and systematic biases in object location memory

**DOI:** 10.3758/s13414-021-02243-y

**Published:** 2021-03-15

**Authors:** Vladislava Segen, Giorgio Colombo, Marios Avraamides, Timothy Slattery, Jan M. Wiener

**Affiliations:** 1 Ageing and Dementia Research Centre, Bournemouth University, Bournemouth, UK; 2 Department of Psychology, Bournemouth University, Bournemouth, UK; 3 ETH Zurich, Future Health Technologies, Singapore-ETH Centre, Singapore, Singapore; 4 Department of Psychology, University of Cyprus, Nicosia, Cyprus; 5 CYENS Centre of Excellence, Nicosia, Cyprus

**Keywords:** Spatial memory, 3D perception: Space Perception, Spatial cognition

## Abstract

The aim of the current study was to develop a novel task that allows for the quick assessment of spatial memory precision with minimal technical and training requirements. In this task, participants memorized the position of an object in a virtual room and then judged from a different perspective, whether the object has moved to the left or to the right. Results revealed that participants exhibited a systematic bias in their responses that we termed the reversed congruency effect. Specifically, they performed worse when the camera and the object moved in the same direction than when they moved in opposite directions. Notably, participants responded correctly in almost 100% of the incongruent trials, regardless of the distance by which the object was displaced. In Experiment 2, we showed that this effect cannot be explained by the movement of the object on the screen, but that it relates to the perspective shift and the movement of the object in the virtual world. We also showed that the presence of additional objects in the environment reduces the reversed congruency effect such that it no longer predicts performance. In Experiment 3, we showed that the reversed congruency effect is greater in older adults, suggesting that the quality of spatial memory and perspective-taking abilities are critical. Overall, our results suggest that this effect is driven by difficulties in the precise encoding of object locations in the environment and in understanding how perspective shifts affect the projected positions of the objects in the two-dimensional image.

**Supplementary Information:**

The online version contains supplementary material available at 10.3758/s13414-021-02243-y.

Our ability to orient and navigate depends largely on forming spatial representations that maintain information about the locations of landmarks and other objects (Epstein et al., 1999; Postma et al., 2004; Waller, 2006). Such representations can vary greatly in terms of the precision with which they hold information (Evensmoen et al., 2013). In the visual working memory literature, the precision of spatial representations has been investigated with tasks that involve memorizing first the position of an object presented in a 2D stimulus array on a blank screen, and then repositioning the object to its original position (Aagten-Murphy & Bays, 2019; Nilakantan et al., 2018; Pertzov et al., 2012; Pertzov et al., 2015; Stevenson et al., 2018). Moreover, psychophysical approaches with change detection tasks have also been used to quantify the precision of spatial representations (e.g., Brady & Alvarez, 2015; Luck & Vogel, 1997, 2013). In these tasks, participants are asked to indicate whether an object has moved or changed between encoding and test, with the amount of movement/change systematically manipulated. Such tasks, which are primarily designed to investigate visuospatial working memory capacity, showed that increasing the number of to-be-remembered items leads to a reduction in the quality of the representation for each of the items (Brady et al., 2011). In addition, the precision of encoding was shown to be negatively affected by typical and atypical aging (Liang et al., 2016; Nilakantan et al., 2018; Pertzov et al., 2015).

Although such approaches provide a detailed account of the precision with which object locations can be memorized, they typically focus on 2D spaces and do not investigate the precision of spatial representations in the 3D space that we encounter during navigation, where perspective shifts typically take place. In addition, tasks that use 2D spaces can often be solved by memorizing the pixel positions of the objects on the screen and thus do not require participants to infer how space is structured (Nardini et al., 2009). In contrast, the use of virtual environments and the introduction of perspective shifts between encoding and test allows investigating the ability to encode 3D spatial locations. It also ensures that participants cannot simply memorize the position of the objects on the screen. Instead, participants must remember the position of the object in the virtual world and understand how the visual projections of the objects and the room would change following a perspective shift.

There are several virtual reality navigation tasks that allow assessing the precision of spatial representations. In some tasks, participants have to learn the position of target locations within an environment—that is, virtual Morris water maze (vMWM) tasks (e.g., Daugherty et al., 2015; Moffat et al., 2007; Woolley et al., 2010), the flag localization task (Hartley et al., 2004), object-location memory tasks (Doeller et al., 2008)—while in other tasks they have to memorize their own locations before being transported to a new location and asked to navigate back to the previous location (e.g., Gillner et al., 2008). These experimental tasks provide rich data sets with a wide range of measures that allow assessing the precision with which spatial locations can be memorized, such as distances and angular differences between the estimated position of the target or own location and the correct locations, time spent searching in the vicinity of the correct location, and path trajectories amongst others. These tasks have also been used to investigate spatial encoding strategies (e.g., Mueller et al., 2008) and reference frames (e.g., King et al., 2002; King et al., 2004) used during navigation as well as effects of (a)typical aging on spatial navigation (e.g., Moffat et al., 2007). More recently, the vMWM has also been applied to investigate the precision of spatial representations in patients with hippocampal lesions (Kolarik et al., 2018; Kolarik et al., 2016).

Despite their utility for studying the precision of spatial memory, these tasks often require specialized equipment, software, and skills, as well as prolonged training and familiarization with the task, the virtual environment, and the equipment. For example, a typical virtual Morris water maze task consists of training trials during which participants learn the position of the hidden platform by navigating within the environment (Daugherty et al., 2015; Kolarik et al., 2018; Kolarik et al., 2016; Moffat et al., 2007; Woolley et al., 2010) as well as control trials where participants navigate to a visible platform. In addition, those tasks require participants to navigate/move within the environment using a keyboard or a joystick, which can introduce unwanted confounds that depend on gaming and computing experience (Murias et al., 2016; Richardson et al., 2011). This becomes a particular challenge if testing involves patients and older adults, who are often less experienced in using such devices (Charness & Boot, 2009; Diersch & Wolbers, 2019). Difficulties with the testing apparatus can inflate differences in navigation performance (Richardson et al., 2011; Waller, 2000). Moreover, the in-depth analysis of performance on those virtual navigation tasks, which is needed to estimate the precision of spatial representations (Kolarik et al., 2018; Kolarik et al., 2016), can often be quite complex (Cooke et al., 2019).

Spatial memory and perspective-taking tasks offer advantages for studying the precision of spatial representations over navigation tasks as they are easier to administer and require neither prolonged training nor specialized equipment. Typically, they involve an encoding stimulus portraying a place or an array of objects that participants have to memorize, followed by the presentation of a second stimulus presented from a different perspective with participants asked to judge whether it depicts the same place, or whether the objects have moved (Hartley et al., 2007; Hilton et al., 2020; Montefinese, Sulpizio, Galati, & Committeri, 2015; Muffato et al., 2019; Segen, Avraamides, Slattery, & Wiener, 2020a).

A popular spatial memory task that follows this paradigm is the Four Mountains task (Hartley et al., 2007), which involves viewing an image of a place defined by four mountains, followed by four new images. One of these images depicts the same place, but from a different perspective, while the other images display a slightly different arrangement of the mountains. Participants are asked to select from the four, the image that corresponds to the same place they have seen during encoding. The Four Mountains task was specifically designed to provide a test that is quick and easy to administer, tapping into viewpoint independent spatial memory. What is more, the task has been successfully used to differentiate between healthy older adults and those with mild cognitive impairment (MCI) as well as between MCI, Alzheimer’s disease (AD), and frontotemporal dementia patients (Bird et al., 2010; Chan et al., 2016).

The Four Mountains task, however, does not systematically manipulate the amount of change of the spatial situation between encoding and test and is therefore not suited to assess the precision of spatial representations. Similarly, spatial memory tasks that focus on object location binding typically either move the object by a specific invariant amount (Montefinese et al., 2015) or swap two objects with each other (Hilton et al., 2020; Muffato et al., 2019; Segen et al., 2020a, 2020b). Again, such manipulations do not allow the assessment of the precision with which spatial locations are encoded.

Spatial memory precision has recently been associated with hippocampal functioning (Ekstrom & Yonelinas, 2020; Kolarik et al., 2018; Kolarik et al., 2016; Stevenson et al., 2018). For example, Stevenson et al. (2018) reports that increased high-frequency activity in the hippocampus was associated with the precision of spatial memory retrieval in a task using 2D stimuli. Moreover, Kolarik et al. (2016) and Kolarik et al. (2018) showed that hippocampal damage was associated with deficits in the ability to precisely remember the position of targets while coarse memory for the targets’ approximate locations was not affected. Importantly, the hippocampus and related regions undergo functional and anatomical changes in typical and atypical aging, which are often associated with declines in spatial memory (Hartley et al., 2007; Hilton et al., 2020; Montefinese et al., 2015; Muffato et al., 2019; Segen et al., 2020a). However, the nature of those deficits is not well understood as the findings reporting deficits are often mixed, specifically in healthy older adults and those with very early MCI (Colombo et al., 2017; Moodley et al., 2015; Segen et al., 2020b). Quantifying the precision of spatial memory may offer a more sensitive tool, compared with studies focusing on coarse spatial changes (Hartley et al., 2007; Hilton et al., 2020; Montefinese et al., 2014; Muffato et al., 2019; Segen et al., 2020a), to investigate spatial memory deficits in those groups. As a result, a quick and accurate tool that taps into the precision of spatial representations would provide a more nuanced understanding of the nature of spatial deficits across those groups—that is, (a)typically aged groups—that could be extended for early detection of MCI as well as differential dementia diagnosis in the future.

Here, we set out to develop a novel spatial memory task that aims to provide a quick and objective assessment of precision of spatial encoding, with minimal training requirements. To do so, we developed a two-alternative forced-choice (2AFC) task where participants had to judge the direction in which an object has moved in a 3D environment following a perspective shift. By systematically manipulating the distance by which the object was displaced, we estimated how accurately participants could detect the movement of objects in space following a perspective shift.

## Experiment 1

### Introduction

In this experiment, we introduce a novel task that was designed to provide a quick assessment of the precision of object location representations in healthy younger adults. We employed psychophysics methods using an 2AFC task in which participants had to judge the direction in which an object moved in an environment following a perspective shift. A 2AFC approach was chosen as it is better suited to rapidly and reliably assess precision of spatial memory than change detection tasks (Heywood-Everett et al., 2020). To investigate the precision of participants’ representations for object locations, we systematically manipulated the distance by which the object was displaced.

### Method

#### Participants

In total, 44 participants between the ages of 20 and 48 (*M*_age_ = 25.5, *SD* = 6.31) years of age took part in the study (29 females; 15 males). The majority of the participants (40) were right-handed. Participants were recruited through Bournemouth University’s participant recruitment system and received monetary compensation for their time. Written informed consent was obtained in accordance with the Declaration of Helsinki.

#### Design

The experiment followed a within 2 (object direction: left/right) × 2 (camera direction: left/right) × 6 (object displacement distance [ODD]: 5, 8, 13, 22, 37, 61 cm) design.

#### Materials

##### Virtual environment

The virtual environment was designed using 3DS Max 2018 (Autodesk Inc.) and consisted of a square room (9.8 m × 9.8 m), on the walls of which there were posters depicting highly familiar and recognizable landmarks (Hamburger & Röser, 2014). A teal plank was placed diagonally in the middle of the room (14-m long), and a target object was placed centrally on that plank with its position varied within a range of 65 cm either to the left or right of the center. The target object could only move along the plank.

**Fig. 1:**
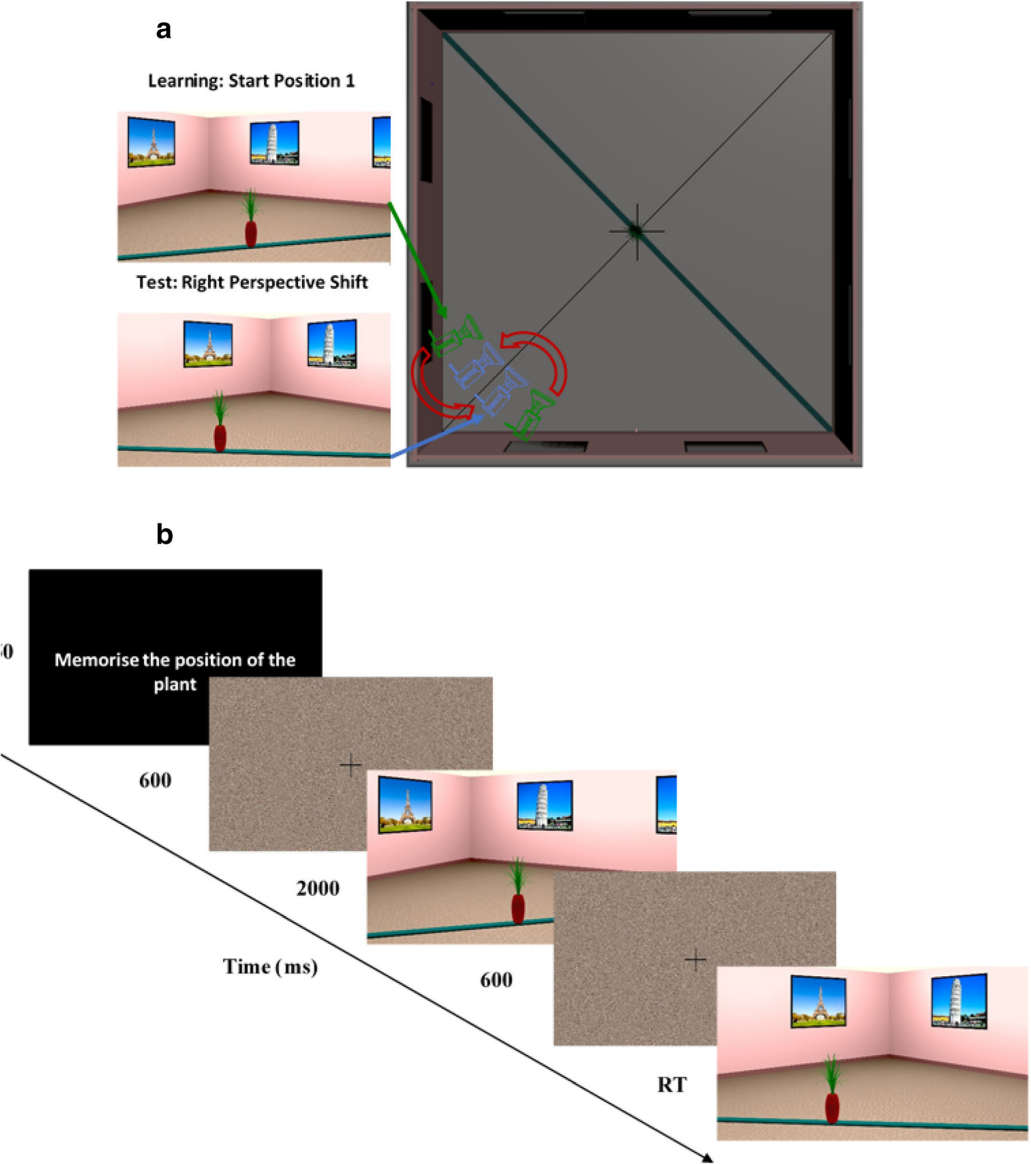
**a** Top-down schematic of the virtual environment used in the experiment with camera positions. Green cameras represent camera positions at encoding, and blue cameras represent the corresponding camera positions at test. Red arrows indicate the direction of perspective shift for each of the encoding cameras. Example renderings from the encoding (Start Position 1) and test camera are also provided. **b** Trial structure. (Color figure online)

The experimental stimuli were renderings of the environment with a 47.7° horizontal field of view and a 15% shift in the vertical field of view to simulate human vision (see Fig. 1a). Creating an asymmetric viewing frustum that resembles natural vision has been found to improve distance estimates in virtual environments (Franz, 2005). The experiment was presented on an 80.9-cm screen (diagonal) with an aspect ratio of 16:9 and a resolution of 1,920 × 1,080 pixels. Participants were seated 80 cm from the monitor with their head positioned on a chin rest. The physical vertical field of view (FOV) of the screen at this distance was 28°, and the horizontal FOV was 47.7° and matched the horizontal FOV of the rendered stimuli.

The cameras were arranged around an invisible diagonal line that was perpendicular to the plank. In both encoding and test stimuli, participants would always see one corner of the room and two posters on either side of the corner (see Fig. 1a). There were two possible camera start and object start positions in encoding stimuli. The two possible camera start positions were 15° to the left (Position 1) or to the right (Position 2) of the diagonal line (see Fig. 1a). The target object was positioned on the plank, either 5 cm to the left or to the right of the center of the room. The camera always faced the center of the room.

The test stimuli were rendered from a different viewpoint with a 20° perspective shift. If the stimuli at encoding was presented from Camera Position 1, the camera moved right, and if the encoding was presented from Camera Position 2, it moved left (see Fig. 1a). The target object at test would move by 5, 8, 13, 22, 37, or 61cm from its start position either to the left or the right.

Stimuli were presented with OpenSesame 3.1.7 (Mathôt et al., 2012), and the left and right arrow keys on a standard computer keyboard were used to record responses.

#### Procedure

Each experimental trial started with a brief presentation of text instructing participants to remember the location of the target object (750 ms), followed by the presentation of a fixation cross and a scrambled stimuli mask (600 ms; see Fig. 1b). In the subsequent encoding phase, participants were presented with a rendering of one of the two target object start positions either from Camera Position 1 or Position 2 for 1.7 seconds. After the encoding phase, participants were again presented with a fixation cross and a scrambled stimuli mask for 600 ms. In the test phase, participants were presented with a rendering of the room following a 20° perspective shift. Their task was to decide whether the target object has moved to the left or to the right and respond by pressing the corresponding key on a standard computer keyboard. In 50% of the trials, the target object moved left, and in the remaining 50% of the trials, the target object moved right.

The experiment consisted of 72 experimental trials presented in randomized order, with each object displacement distance repeated eight times. The task took around 10–15 minutes to complete and was administered as part of a larger study.

### Results

Accuracy estimates were obtained using generalized linear mixed-effects(GLME) models using the *glmer* function from LME4 package (Bates, Kliegl, Vasishth, & Baayen, 2015) in R, with ODD (object displacement distance), camera direction, and object direction as fixed factors and a random by-subject and by-stimuli intercept. We also estimated corresponding *p* values using the lmerTest package (Kuznetsova, Brockhoff & Christensen, 2017). Both camera direction and object direction were effect coded, and ODD was scaled and log transformed and used as a continuous variable. Our results (see Table 1) showed that performance increased with an increase in the distance by which the target object was displaced between encoding and test. In addition, we found an interaction between camera direction and object direction, with lower performance in situations when the camera and the object moved in the same direction (e.g., the target object moves left, and the camera moves left). We also found a three-way interaction between camera direction, object direction, and ODD, in which the effect of camera and object direction was reduced with an increase in the ODD.

**Table 1:** Coefficients from accuracy GLME analysis

Predictors	Accuracy			*p* values
	Coefficients	*SE*	*z* value	
(Intercept)	1.428	0.183	7.787	**< .001**
Camera direction (left)	0.045	0.072	0.620	.535
Object direction (left)	0.076	0.072	1.052	.293
Log (ODD)	0.837	0.074	11.327	**< .001**
Camera Direction × Object Direction	− 1.965	0.078	− 25.167	**< .001**
Camera Direction × Log (ODD)	− 0.062	0.072	− 0.858	.391
Object Direction × Log (ODD)	0.034	0.072	0.467	.640
Object Direction (Left) × Camera Direction × Log (ODD)	0.393	0.074	5.335	**< .001**

Significant *p* values (|*p*| ≥ 0.05) in bold

#### Reversed congruency effect

**Fig. 2:**
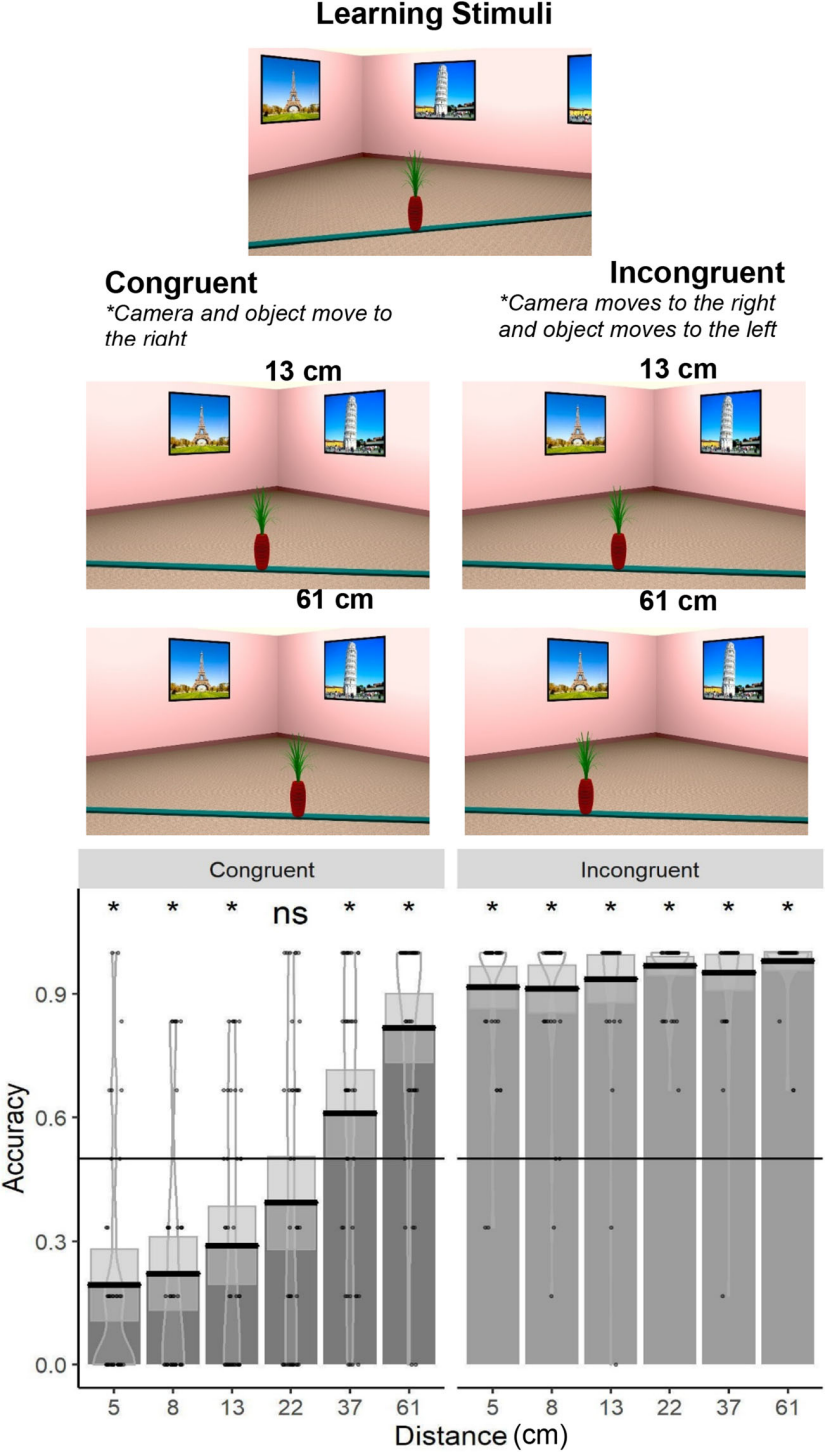
The upper panel shows example stimuli from the learning and the test phases for congruent and incongruent trials across 13 cm and 61 cm object movements. The bottom panel shows accuracy as a function of distance (cm) and congruency, with mean (solid line) and 95% CIs (gray shaded area) and individual data points and violin plots behind. Chance level performance is indicated by the solid horizontal line

To further investigate the Camera × Object Direction interaction, we split data into *congruent* and *incongruent* trials. In congruent trials, the camera and the object moved in the same direction, whereas in incongruent trials, the camera and the object moved in opposite directions. We then ran a GLME to investigate the effect of congruency and ODD on performance. The same random effect structure was used as in the previous analysis. The results (see Table 2) show that participants performed significantly worse in congruent trials than in incongruent trials, and we termed this bias the reversed congruency effect. We also found a two-way interaction with the reduction of the reversed congruency effect with an increase in distance (see Fig. 2). Specifically, our results show that in the congruent trials, participants consistently reported that the object moved in the opposite direction of the actual movement for small displacements (i.e., 5cm–22 cm). Only once the object was moved by 37 cm or more (61 cm), participants began to perform above chance level in the congruent trials (see Fig. 2). A different pattern of results was found in incongruent trials, with participants responding correctly on more than 90% of the trials, regardless of the ODD.

**Table 2:** GLME analysis investigating the congruency

Predictors	Accuracy			*p* values
	Coefficients	*SE*	*z* value	
(Intercept)	1.458	0.186	7.849	**< .001**
Log (distance)	.852	0.079	10.807	**< .001**
Congruency (congruent)	− 1.910	0.082	− 23.174	**< .001**
Log (distance) × Congruency (congruent)	0.370	0.079	4.705	**< .001**

Significant *p* values (|*p*| ≥ 0.05) in bold

### Discussion

This experiment set out to establish a new task that allowed for a quick and easy assessment of the precision of spatial representations. Unexpectedly, we found that the combination of object and camera movement direction systematically biased participants' responses. Specifically, if the object and the camera moved in the same direction, participants perceived the movement of the object to be in the opposite direction. This was most pronounced at smaller displacement distances. If, however, the object and the camera moved in the opposite directions, participants reliably detected movement direction, even at the smallest displacement distances. We termed this the reversed congruency effect.

It is not obvious how spatial cognition theories, including those differentiating between egocentric and allocentric spatial representations (Burgess, 2006; Klatzky, 1998; Shelton & McNamara, 2001), could explain this reversed congruency effect. For example, if participants formed an allocentric representation of the environment (Burgess, 2008), they should reliably detect the direction of object movement regardless of whether the camera and the object moved in the same or opposite directions. This is because their representations of object locations are encoded relative to other features or landmarks in the environment and do not depend on the perspective from which the environment is viewed. Similarly, if participants encode the position of the object and other environmental cues in relation to their current position in space and engage in mental transformations to achieve spatial perspective taking (Holmes et al., 2018; King et al., 2002; Klencklen et al., 2012), we would expect them to adjust the expected positions of the objects in the environment based on their new position in the environment and perform the task without the systematic bias that we observed. Of course, neither the egocentric nor the allocentric strategy would guarantee that participants always responded correctly. Instead, performance would depend on the individual’s ability to generate precise spatial representations. Thus, we expected a linear increase in performance in both congruent and incongruent trials with increasing target object displacement, with the slope and intercept of the increase being determined by individual differences in precision.

If participants, as argued above, did not solve the task using a spatial strategy (i.e., egocentric or allocentric strategy), it is possible that they used a heuristic that may have given rise to the systematic bias we have observed. We considered a number of such heuristics for the reversed congruency effect (more information on those heuristics is available in the Supplementary Materials). First, given the relatively small extent of the camera movement between encoding and test, participants may have found it difficult to understand the perspective shift and, therefore, essentially ignored it. As a result, they would have remembered the position of the target object on the screen (i.e., in screen coordinates) and used this position to judge whether the object has moved to the left or right. The screen-based strategy would be akin to participants using an egocentric strategy that would ignore the perspective shift and use the absolute relationships between their body and the object to judge the direction in which the object has moved. This screen-based strategy, however, predicts correct response for all trials, a pattern that we did not observe in the congruent trials. Second, participants could have encoded the position of the target object relative to other room-basedcues—such as the room corner—but in the image, rather than in the 3D space. During test, they may have compared this memorized relationship with that in the test image in order to decide whether the object moved left or right. This “corner-based” strategy does predict correct responses in all incongruent trials, thus predicting participants’ performance well in these trials. However, the corner-based strategy predicts incorrect responses for all congruent trials, which does not match the empirical data.

We believe that the reversed congruency effect is primarily driven by the movement of the camera in the real world such that when the camera moves left, participants expect that the object would appear to move left as well. As a result, even if the object remained stationary, participants would experience “camera-induced object motion” to the right (as they expected that it would move to the left). This camera-induced object motion, together with actual object movement direction, would give rise to the reversed congruency effect. Specifically, if the object moves in the opposite direction to the camera (incongruent trials), the camera-induced object motion amplifies the actual object movement. In contrast, when the object moves in the same direction as the camera (congruent trials), the camera-induced object motion effect may be greater than the actual object movement. In such cases, participants would incorrectly perceive the direction of object movement. However, when the object movement is large enough, it will eliminate the induced motion effect caused by the camera movement, and participants may perceive the object movement in the correct direction. This interpretation is in line with our empirical data, as participants consistently misjudged the direction of movement for small object displacements in congruent trials with performance improving for larger displacements. In incongruent trials, on the other hand, participants responded correctly across all object displacement distances.

To our knowledge, there have been no other reports that have described an “induced object motion effect” after a perspective shift in the spatial cognition literature. We did, however, find reports from studies with 2D stimuli that describe an induced object motion effect, called the induced Roelofs effect (IRE; Bridgeman et al., 1997). Specifically, when a dot and a surrounding rectangular frame move in opposite directions on the screen, participants perceive the movement of the dot as larger than when the dot and the frame move in the same direction (Abrams & Landgraf, 1990; see also, Bacon et al., 1982). The IRE has also been demonstrated using static stimuli showing that if the frame is shifted to the left, participants estimate the target object to be farther to the left (Bridgeman et al., 1997; Taghizadeh & Gail, 2014). Two explanations have been proposed for the IRE: (1) the frame biases the egocentric perceived midline in the direction of the frame shift, thus changing the location of the target relative to perceived midline (Bridgeman et al., 2000; Bridgeman et al., 1997); (2) the effect is induced by an allocentric influence with the relative relationship between the target and the frame directly affecting the perceived location of the target (de Grave et al., 2002; Taghizadeh & Gail, 2014). Importantly, both explanations suggest that the IRE stems from biased encoding as a result of the shift of the frame position on the screen. In our experiment, it is not clear what the frame would be as the stimuli were always presented full screen and thus did not move on the screen. Thus, the camera-induced object motion effect in our experiment is unlikely to be driven by the same mechanisms that describe the IRE. Instead, we propose that the camera-induced object motion is the product of the camera movement in the “real world” (virtual environment) between encoding and test rather than by the movement of the object on the screen. While we do not have a firm explanation for the camera-induced object motion effect we observed, we speculate that it is driven by difficulties in precisely encoding the position of the object in the environment and difficulties in understanding how the perspective shift between encoding and test affects the projected position of the object in the two dimensional image. It is also possible that the camera-induced object motion effect experienced by participants may arise due to naïve theories that people hold about how the visual world works (for more in-depth discussion, see Bertamini, Latto, & Spooner, 2003). It is also worthwhile to note, that the encoding time was relatively short, as a result it is possible that this has contributed to difficulties in precisely encoding object position.

The primary aim of Experiment 1 was to introduce a new task to assess the precision with which participants can memorize the locations of objects in space. The reversed congruency effect described above, however, demonstrates that the perspective shift between encoding and test had a significant impact on participants’ judgments. Therefore, Experiment 2 was designed to facilitate our understanding of the reversed congruency effect. Specifically, we investigate whether the effect is driven by object movement on the screen or as a result of camera movement in the real world. Experiment 2 aimed to provide a conceptual replication of the reversed congruency effect, but also aimed to investigate whether providing additional cues in the environment would eliminate or at least reduce the effect.

## Experiment 2

### Introduction

This experiment set out to further investigate the reversed congruency effect observed in Experiment 1. We proposed that the effect is driven by movement of the camera in the virtual environment that induces object motion. However, in Experiment 1, the object position on the screen differed between encoding and test, and thus the object moved both on the screen as well as in the virtual environment. In Experiment 2, we held the object position on the screen constant between encoding and test, thus allowing us to investigate whether the reversed congruency effect described above was driven by the object movement in the virtual environment or on the screen.

Difficulties in forming a precise spatial representation of object positions in the environment are likely to increase susceptibility to the bias induced by camera movements that give rise to the reversed congruency effect. To reduce both the proposed induced object movement based on the perspective shift and the susceptibility to the induced movement based on uncertainty in the memorized object location, we introduced additional objects in the environment both during encoding and test. We expected that enriching the spatial scene with these additional objects would help participants to better memorize the exact object location (Cánovas et al., 2011; Chamizo et al., 2011) and to understand the perspective shift.

In addition, we have increased the encoding time, as we thought that the short encoding times in Experiment 1 could have prevented participants from formulating precise representations of object positions. Lastly, due to COVID-19 restrictions that prevented in-person laboratory testing, Experiment 2 was carried out online. Based on recent research indicating that online data collection can provide valid and reliable data on a variety of cognitive and perceptual experiments (Huber & Gajos, 2020; Komarov et al., 2013; Reinecke & Gajos, 2015), we expected to replicate the reversed congruency effects of Experiment 1, in the condition without additional objects, as it was most similar to that of Experiment 1.

### Method

#### Participants

Forty-seven participants (40 females and seven males) between 18 and 35 years old (*M*_age_ = 21.94, *SD* = 4.09) took part in the study. Participants were recruited through Bournemouth University’s participant recruitment system and through online advertising. All participants provided their informed consent in accordance with the Declaration of Helsinki.

#### Materials

##### Virtual environment

**Fig. 3:**
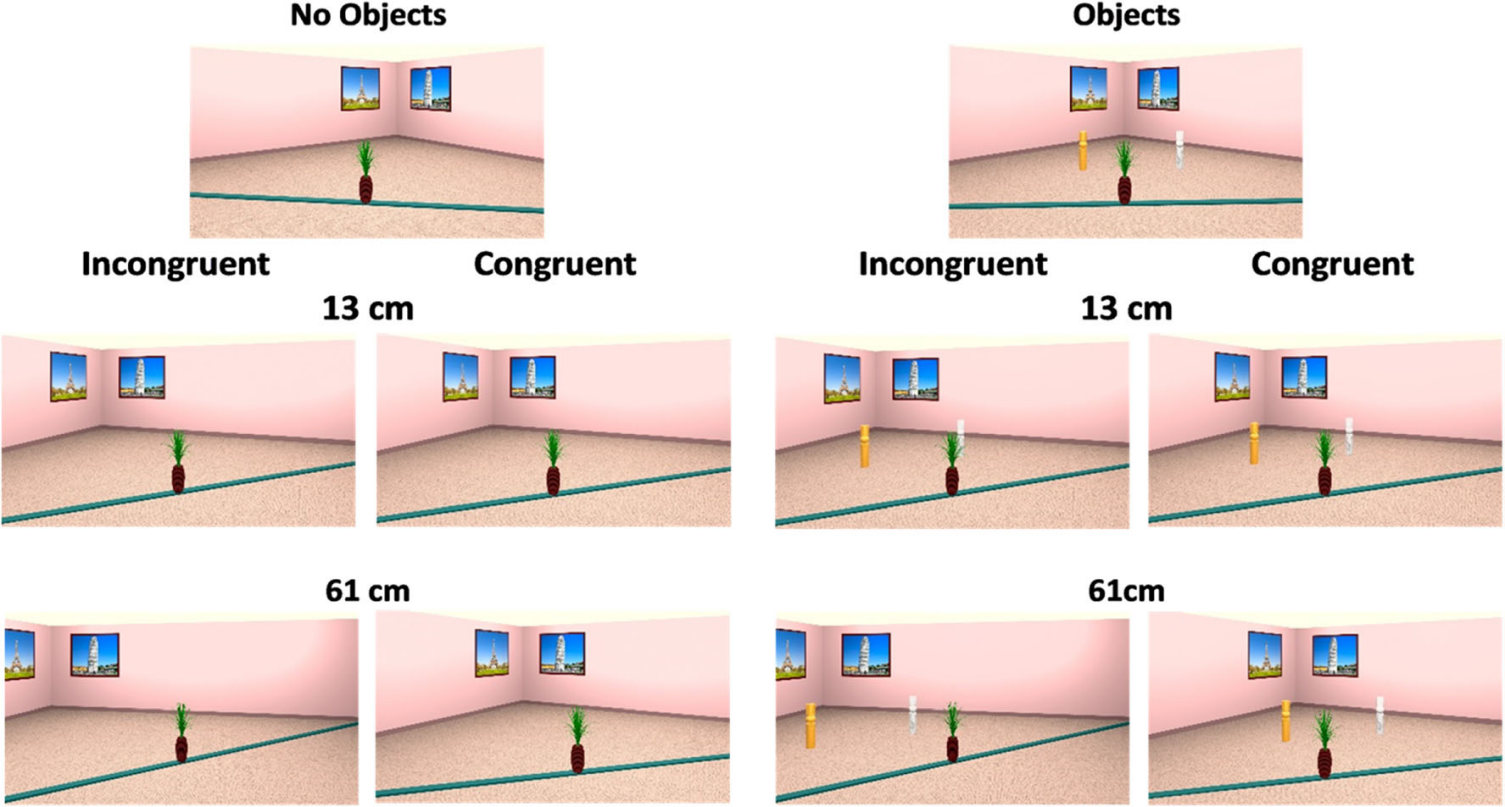
Stimuli examples for the no objects and additional objects in incongruent and congruent trials for a 13 and 61 ODD

We used the same virtual environment as in Experiment 1. However, in this version of the task the camera was always directed such that the target object was in the center of the screen, regardless of the position of the camera and the target object within the environment. Thus, in order to solve the task participants were required to consider the movements of the object in the world rather than on the screen. In addition, the camera moved by 20 degrees between encoding and test either to the left or the right, regardless of the camera position during encoding. This ensured that the camera position during encoding could not be used to predict its movement, as was the case in Experiment 1. We also added the additional-objects condition, in which two view-invariant columns were added to the environment (see Fig. 3). The columns were placed approximately halfway between the walls and the plank, such that they were closer to the target object than the posters that were available in Experiment 1 and the no-additional-object condition. The columns were positioned at equal distances either to the left or to the right of the center of the room on the horizontal plane. The target object at test could move by either 8, 13, 22, 37 or 61 cm from the start position, either in the left or right direction on the plank.

#### Design

The experiment followed a within 5 (object displacement distance [ODD]: 8, 13, 22, 37, 61 cm) × 2 (condition: additional objects/no objects) × 2 (congruency: congruent/incongruent) design.

#### Procedure

Participants completed the task using an online testing platform Testable (testable.org). Prior to the experiment, they were presented with instructions on how to calibrate their screen to ensure that the entire stimulus was visible during the experiment which was run in full screen mode. The experimental setup was similar to Experiment 1; however, encoding times were increased from 1.7 s, as in Experiment 1, to 5 s in Experiment 2.

The experiment began with four practice trials that preceded the 160 experimental trials. The additional-object manipulation was blocked and counterbalanced such that half of the participants completed the no-object condition first and the other half completed the additional-object condition first. Within each block, trials were presented in randomized order, with the entire experimental procedure taking around 35 minutes to complete.

### Results

To investigate performance, we ran a GLME with ODD, additional objects, and congruency as fixed factors and random by-subject and by-stimuli intercept and slope for condition. Results showed that accuracy increased with the increase in the ODD (see Table 3). Consistent with our predictions, we replicated the reversed congruency effect reported in Experiment 1, with lower performance in congruent compared with incongruent trials. We also found that accuracy was lower in the no-objects condition than in the additional-objects condition. Importantly, we found a Congruency × Condition interaction, whereby the reversed congruency effect was no longer a reliable predictor of performance in the additional-objects condition (see Supplementary Materials for follow-up analysis). Lastly, we found a two-way interaction between ODD and condition, with a larger increase in performance in the additional-objects condition when ODD increased.

**Table 3:** Coefficients from accuracy GLME analysis

Predictors	Accuracy			*p* value
	Coefficients	*SE*	*z* value	
(Intercept)	0.329	0.027	12.044	**< .001**
Log (ODD)	0.256	0.027	9.344	**< .001**
Congruency (congruent)	− 0.404	0.027	− 14.799	**< .001**
Condition (additional objects)	0.135	0.032	4.217	**< .001**
Log (ODD) × Congruency (congruent)	0.038	0.027	1.387	.165
Log (ODD) × Condition (additional objects)	0.070	0.027	2.555	**.011**
Congruency (congruent) × Condition (additional objects)	0.239	0.027	8.773	**< .001**
Log (ODD) × Congruency (congruent) × Condition (additional objects)	0.023	0.027	0.842	.400

Significant *p* values (|*p*| ≥ 0.05) in bold

We also ran a GLME model with additional fixed effects (block and camera rotation). We found some order effects such that the increase in performance in the additional-objects condition was higher in those who have completed the additional-objects condition first. This interaction was most likely driven by a larger increase in performance in incongruent trials in the additional-objects condition, when this condition was performed first. The complete model is reported in the supplementary materials.

### Discussion

In Experiment 2, we replicated the reversed congruency effect of Experiment 1 in the condition without additional objects in the environment. Thus, the reversed congruency effect seems to be robust across different encoding times (1.7 s vs. 5.0 s) and means of data collection (laboratory vs. online). Importantly, the replication of the reversed congruency effect suggests that the effect was not driven by the movement of the object on the screen as the object always remained in the center of the screen in Experiment 2. Instead, the bias likely arises from camera movements that results in an induced object motion in the virtual environment.

The use of additional objects in the environment reduced the reversed congruency effect such that it no longer reliably predicted performance. We believe that the presence of additional objects in the environment helps to reduce the camera-induced object motion effect that gives rise to the congruency effect by doing two things. First, the addition of objects can improve the precision with which object positions are represented. Indeed, past studies show that increased availability of cues/landmarks is associated with more precise spatial encoding (Ekstrom & Yonelinas, 2020; Kamil & Cheng, 2001). This is also consistent with Ekstrom and Yonelinas (2020) who have proposed that the complexity of the environments is associated with the precision of representation, such that less complex environments are encoded using a coarser representation, whilst more complex environments with more features are encoded more precisely. Thus, the additional-object condition may foster a more precise representation by increasing the complexity of the environment such that it contains enough detail that allows participants to understand the precise position the object occupies within the environment.

Furthermore, the additional objects in the current experiment were positioned closer to the target object, compared with the remaining cues (geometric cues and posters) that were available in Experiment 1 and the no-object condition. The proximity of the cues also makes it easier to anchor the target object to those cues. This is in line with previous reports suggesting that use of proximal cues improves memory for target locations (Cánovas et al., 2011; Chamizo et al., 2011).

Second, the addition of objects can also improve spatial perspective taking by providing (1) additional cues that can be used for self-localization following a perspective shift and (2) direct feedback on how perspective shifts affect the 2D projection of the positions that objects occupy on the screen. This feedback can be used to adjust the “expectations” that participants have about where objects are following a perspective shift. For example, if participants expect the objects to move more to the right when the camera moved to the right, but at test they see that the stable cues provided by the additional objects did not “move” as they expected, they can use this information to adjust their expectations for the position of the target object. In addition, the objects in the environment act as additional monocular depth cues that improve depth perception (Luo et al., 2007). Improved depth perception is likely to facilitate the encoding of object positions as well as the understanding of perspective shifts.

## Experiment 3

### Introduction

One of our original aims that motivated Experiment 1 was to design a task that could be used with older adults as a quick measure of the precision of spatial encoding following perspective shifts. Given the results from Experiment 2, where the addition of objects in the scene substantially reduced the reversed congruency effect, we created a shorter variant of the task with additional objects. The main aim of Experiment 3 was to investigate performance differences between younger and older adults and to examine whether the task we designed is suitable to assess the precision of spatial encoding following perspective shifts in older adults.

Although previous research focusing on spatial memory across different viewpoints did not directly investigate precision of spatial representations in older adults. Nevertheless, such studies show age-related deficits in spatial memory across viewpoints (Hartley et al., 2007; Hilton et al., 2020; Montefinese et al., 2015; Muffato et al., 2019) and greater difficulties in older adults to extract useful information from the stimuli after perspective shifts when fine-grained spatial changes were introduced (Segen et al., 2020a).

Additional evidence for age-related declines in precision of spatial encoding comes from 2D tasks used in visuospatial working memory research (Nilakantan et al., 2018; Pertzov et al., 2015). It is plausible that those age-related difficulties in the formation of fine-grained spatial representations are caused by age-related changes in the anterior and posterior hippocampus. Indeed, a recent longitudinal study (Langnes et al., 2019) found that the posterior hippocampus, typically associated with fine-grained spatial processing, was more affected by aging than the anterior hippocampus was, which is involved in the formation of coarser spatial representations (Evensmoen et al., 2013; Nadel et al., 2013).

Age-related functional and morphological changes (Antonova et al., 2009; Meulenbroek et al., 2004; Moffat et al., 2007) in the hippocampal circuit and the retrosplenial cortex may also contribute to spatial perspective taking deficits (King et al., 2002; Vargha-Khadem et al., 1997). However, research investigating how aging affects spatial perspective taking renders mixed results with studies reporting similar effects of perspective shifts on performance in young and older adults on spatial memory tasks (e.g., Hilton et al., 2020; Muffato et al., 2019; Watanabe & Takamatsu, 2014). Other studies report specific age-related deficits in perspective-taking abilities (Inagaki et al., 2002; Montefinese et al., 2015; Segen et al., 2020a; Watanabe, 2011), with older adults being more affected by the presence of the perspective shift rather than its size (Montefinese et al., 2015; Segen et al., 2020a).

Given the age-related declines in spatial memory and precision of spatial encoding across 2D stimuli together with possible perspective-taking deficits, we predicted that older adults would form less precise spatial representations compared with younger adults, which would be reflected in overall lower performance, particularly when the object displacement is small. However, given our current interpretation that the imprecise encoding of the target object position and difficulties in spatial perspective taking contribute to the reversed congruency effect, it is possible that older adults will be more susceptible to the reversed congruency effect.

### Method

#### Participants

Forty young (*M*_age_ = 25.40 years, *SD* = 5.34; age range: 18–35 years; 24 females and 16 males) and 40 older adults aged 55 years and over (*M*_age_ = 63.60, *SD* = 5.17, age range: 55–74; 24 females and 16 males) took part in this study. All participants were recruited through Prolific (https://www.prolific.co), an online participant recruitment system. Participants received monetary compensation for their time. All participants gave their written informed consent in accordance with the Declaration of Helsinki.

#### Materials and procedure

This experiment was similar to Experiments 1 and 2. However, in the current experiment, we only used the environment with additional objects (see additional-objects condition in Experiment 2). Given the overall low performance in Experiment 2 and the prediction that older adults would have less precise spatial memory, we increased the object displacement distance (ODD). Specifically, the target object could move in eight equally sized steps between 10 and 100 cm (10, 23, 36, 49, 61, 74, 87, 100 cm) from the start position, either to the left or right. The camera and object positions were the same as in Experiment 2; however, instead of fixating on the target object as in Experiment 2, the camera always fixated at the center of the room. This was done in order to allow larger ODD whilst ensuring that the same cues were visible at encoding and test.

The experiment consisted of 128 experimental trials presented in randomized order, with 16 trials per ODD. In addition, we included four vigilance trials at random intervals, to ensure that participants were paying attention. In these trials, participants were asked to indicate the side of the screen in which the Eiffel Tower or the Leaning Tower of Pisa poster appeared on. Our criterion of including only data in which participants responded correctly to 3 out of the 4 vigilance trials resulted in no exclusions. The study took around 25 minutes to complete.

### Results

**Fig. 4:**
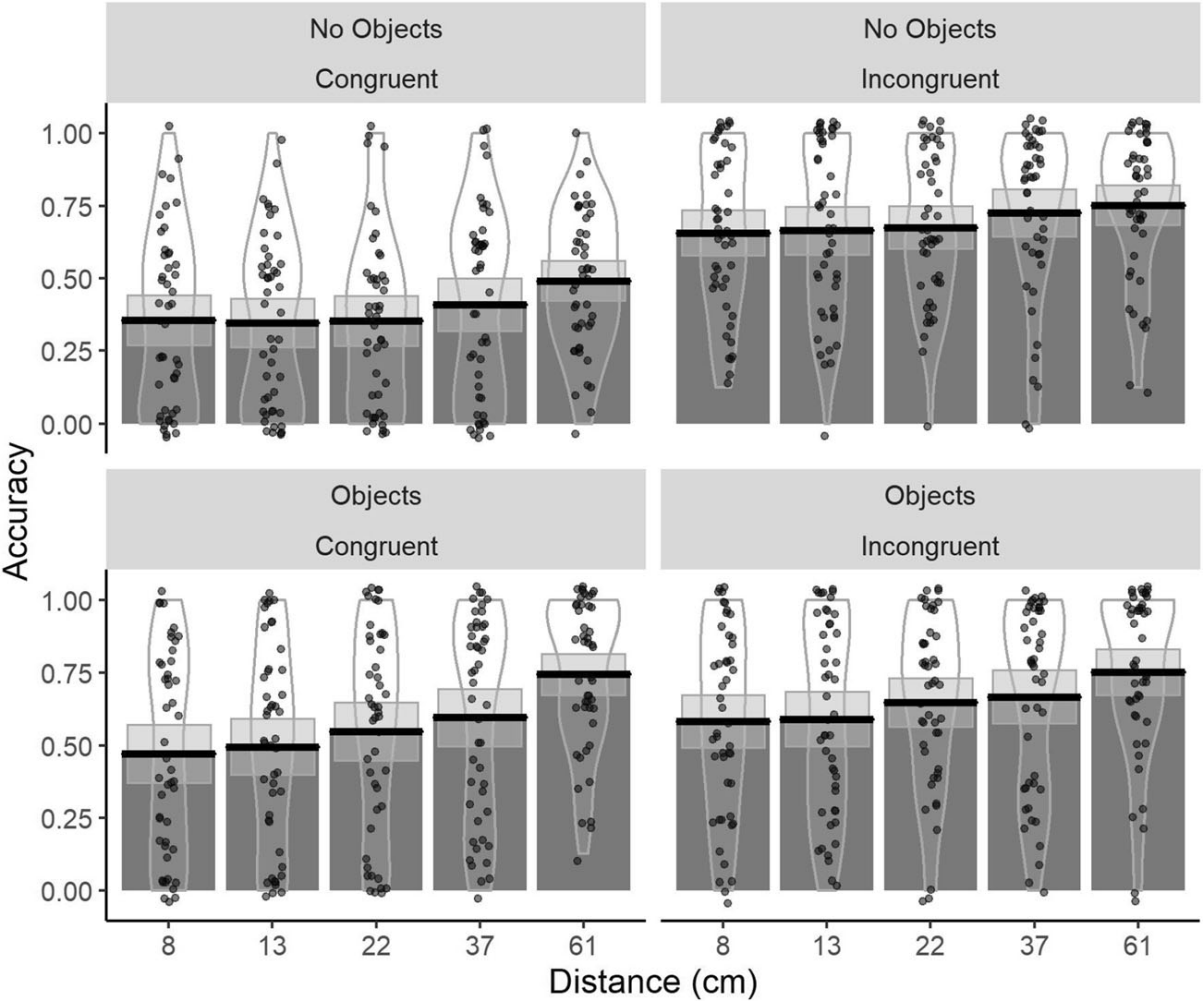
Bar plots for accuracy values as a function of congruency (incongruent/congruent) and condition (no objects/additional objects) and age group, with mean (solid line) and 95% CIs (gray shaded area) with individual data points and violin plots behind

**Fig. 5:**
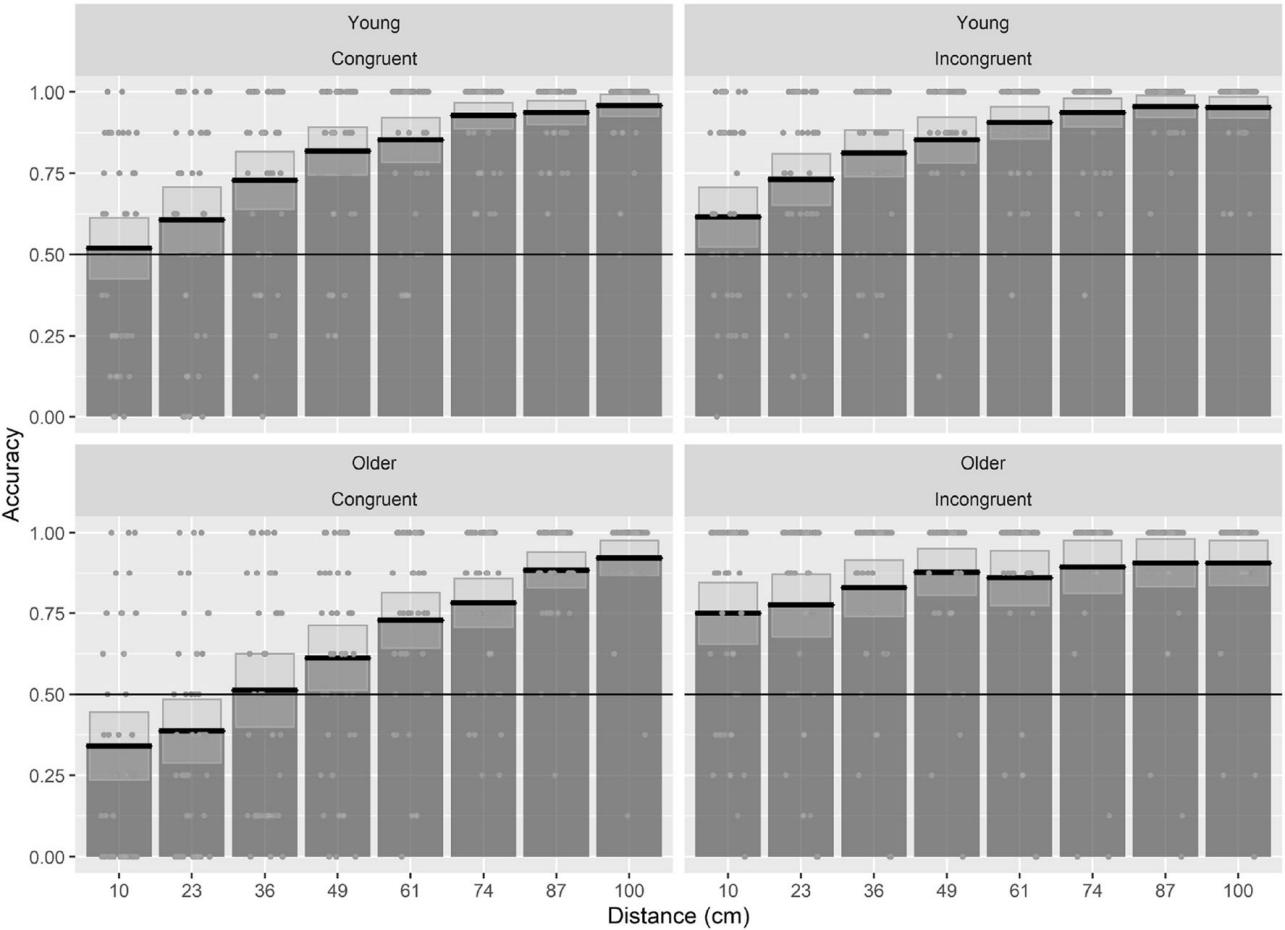
Bar plots for the accuracy as a function age group (young/older adults) and congruency (congruent/incongruent), with mean (solid line) and 95% CIs (gray shaded area) with individual data points. The solid horizontal black line indicates chance level performance

The data was analyzed using GLME, with age group, congruency, and ODD as fixed factors and a random by-subject and by-item intercept. Age group (younger adults/older adults) and congruency (incongruent/congruent) were coded using effect coding. ODD was centered and scaled and used as a continuous variable. We found that age group, congruency, and ODD were all reliable predictors of performance (see Table 4). Specifically, performance increased with increasing ODD, performance was lower in older than in younger adults, and in congruent compared with incongruent trials. We also found a significant interaction between ODD and age group, with a lower increase in performance in our older adults with the increase in ODD. There was also an interaction between ODD and congruency, with a larger increase in performance in congruent trials with increasing ODD. In addition, we found an Age Group × Congruency interaction, with lower performance in older than younger adults in congruent trials. This demonstrates that the reversed congruency effect was larger in the older age group. Lastly, we found a three-way interaction between ODD, age group, and congruency, with older adults showing a larger increase in performance in the congruent trials with increasing ODD (Figs. 4 and 5).

**Table 4:** GLME coefficient for accuracy analysis

Predictors	Accuracy			*p* values
	Estimate	*SE*	*z* value	
(Intercept)	1.756	0.104	16.837	**< .001**
ODD	0.963	0.049	19.683	**< .001**
Age group (old)	− 0.270	0.098	− 2.775	**.006**
Congruency (congruent)	− 0.391	0.048	− 8.191	**< .001**
ODD: Age group (old)	− 0.113	0.032	− 3.523	**< .001**
ODD: Congruency (congruent)	0.236	0.049	4.856	**< .001**
Age Group (old): Congruency (congruent)	− 0.224	0.032	− 7.101	**< .001**
ODD: Age group (old): Congruency (congruent)	0.139	0.031	4.417	**< .001**

Significant *p* values (|*p*| ≥ 0.05) in bold

#### Clustering

Our results demonstrate a lot of variability in performance in both congruent and incongruent trials, which could be driven by individual differences in the strategies used to solve the task. To determine whether there were separate groups of participants who show reliably different performance patterns, we performed a k-means cluster analysis. The cluster analysis was performed on the accuracy data, which was averaged per participant separately for congruent and incongruent trials. To determine the optimal number of clusters, we used the *fviz_nbclust* function (from the factoextra package in R), which calculates the total within cluster sum of squares across a different number of clusters ranging from 1 to 10 across 1,000 iterations. The optimal number of clusters for this data set was 4, as indicated by the elbow method.

**Fig. 6:**
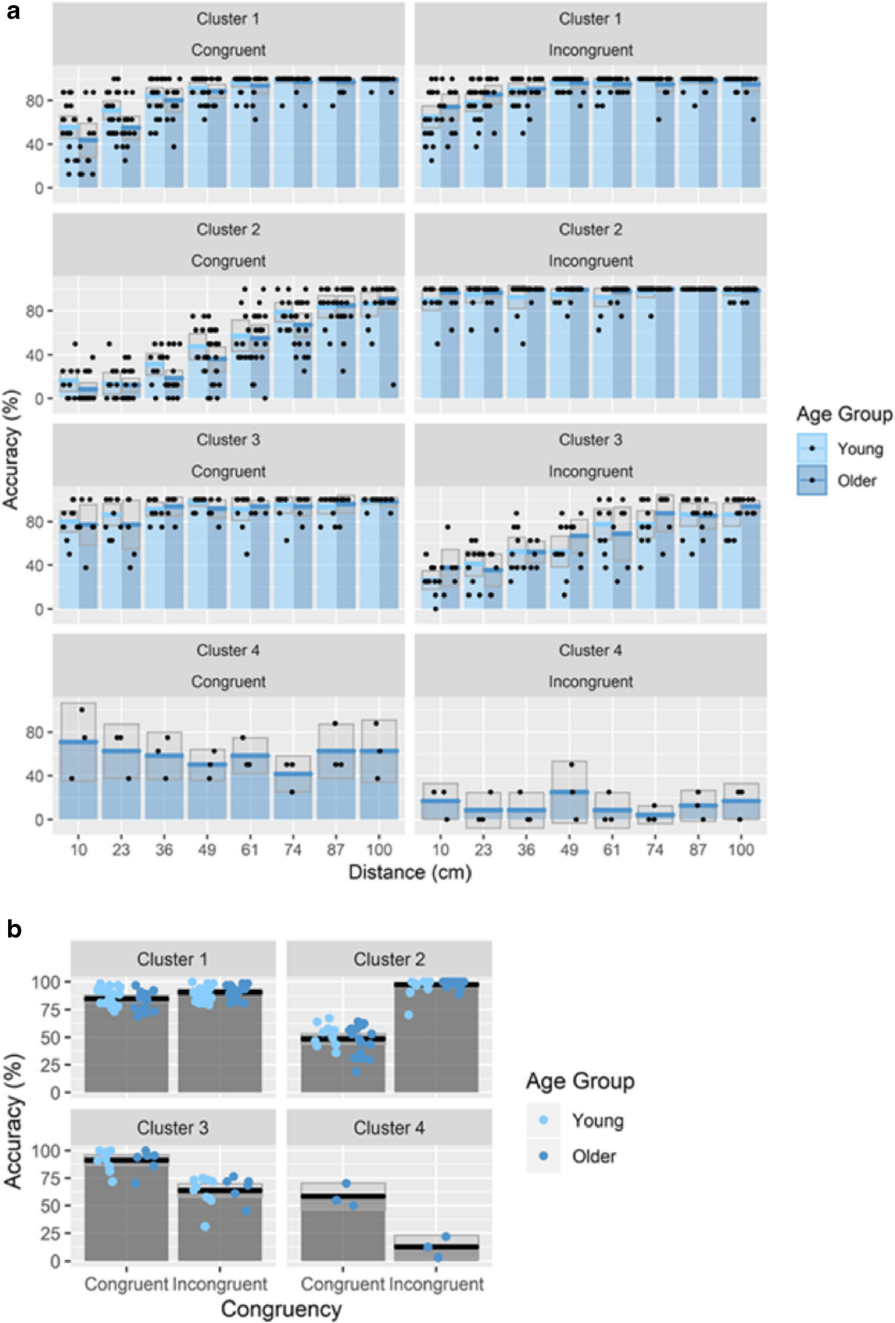
**a** Bar plots for the accuracy for each cluster as a function of congruency and ODD, with mean (solid line) and 95% CIs (gray shaded area) of individual data points. **b** Mean accuracy in each cluster in congruent and incongruent trials with individual data points

The largest group identified by the k-means cluster analysis (Cluster 1) contained 40% of our sample. They performed well across both congruent and incongruent trials (see Fig. 6a–b). Cluster 2 consists of participants whose performance closely resembles the reversed congruency effect found in Experiments 1 and 2, with largest differences between congruent and incongruent trials at smaller ODD (see Fig. 6a–b). This group contained 36.25% of our sample. Cluster 3, made up of 20% of the sample, consisted of participants who showed the “opposite” reversed congruency effect, with higher performance in congruent compared with incongruent trials (see Fig. 6a–b). Finally, in the last cluster, there were only three older participants with low overall performance, which was also not affected by ODD. It is likely that these participants did not understand the task.

Next, we focused on whether the distribution of participants in those clusters varies as a function of age group.^1^ To investigate this, we conducted a chi-squared test with simulated *p* values based on 1,000 iterations. We found that the distributions across clusters were not equal (*p* = .031). For example, almost half of our older participants fell within a cluster in which participants displayed the reversed congruency effect (Cluster 2). On the other hand, half of our young participants fell within the cluster with overall high performance and minimal reversed congruency effect (Cluster 1; see Fig. 6c).

### Discussion

In line with our predictions, we found overall better performance in younger than older adults as well as a larger reversed congruency effect in older than in younger adults. Importantly, our results demonstrated large variability in the pattern of performance for congruent and incongruent trials across participants. To further investigate these individual differences, we used a data-driven clustering approach that identified four distinct clusters of participants based on their performance in congruent and incongruent trials. Specifically, the cluster analysis identified a group that consisted of a large proportion of our sample (40% of participants) who did not show a reversed congruency effect. The fact that this group showed very good overall performance (>80%) demonstrates that participants in this group formulated precise representations of object locations and understood the perspective shifts.

We also found a second large group of participants (~36% of participants) who displayed a reliable reversed congruency effect—specifically, having high performance in incongruent trials and low performance for small object displacement distances in congruent trials that improved as the displacement distances increased. In line with our interpretation of the nature of the reversed congruency effect in Experiments 1 and 2, we believe that this finding indicates that this group had greater difficulties with formulating precise spatial representations and understanding perspective shifts even when additional cues are available in the environment.

Lastly, our clustering analysis identified a group (consisting of 20% of participants) that showed the “opposite” reversed congruency effect. We do not currently understand what may give rise to this performance pattern. Further investigation is needed to understand what drives this “opposite” effect.

Consistent with our prediction that older adults should be more susceptible to the reversed congruency effect, there were more older adults in the group that showed the reversed congruency effect, whilst the reverse pattern was found in the group that did not display the reversed congruency effect, which contained more young adults. Specifically, we hypothesized that older adults would form less precise representations of object locations as result of age-related functional and morphological hippocampal changes (Antonova et al., 2009; Meulenbroek et al., 2004; Moffat et al., 2007). It is also possible that older adults have more difficulties than young adults in understanding perspective shifts (Montefinese et al., 2015; Segen et al., 2020a). As a result of those difficulties, older adults should be more susceptible to the camera-induced object motion, which may give rise to the reversed congruency effect.

In addition, younger and older adults may rely on different spatial strategies to solve the task, as our previous research shows that older participants rely more on cues during spatial memory tasks than younger participants (Segen et al., 2020a, 2020b). Thus, it is possible that if we added even more landmarks/cues into the environment that age differences would be less pronounced.

Overall, the greater tendency of older adults to display the reversed congruency effect is in line with our interpretation that the effect is driven by difficulties in encoding spatial information and understanding the perspective shifts.

## General discussion

In this study, we set out to create a quick and easy to administer task to assess the precision of spatial representations. However, in Experiment 1, we found strong influence of camera and object movement direction, that we termed the reversed congruency effect. Specifically, we found that when the camera and object moved in the same direction (*congruent* movement), performance in identifying the direction of object movement was below chance level for small object displacements. In contrast, performance was at ceiling across all object displacement distances when the camera and the object moved in different directions (*incongruent* movement). This reversed congruency effect was unexpected. In Experiment 2, we established that the reversed congruency effect was driven by the object and camera movement in the virtual world and was not an artifact of the object movement in screen coordinates. If indeed the effect is driven by object and camera movement in the virtual environment, this would demonstrate some egocentric influences in the current task. Specifically, if participants relied solely on an allocentric object-to-object representation, perspective shifts should not introduce any systematic biases in participants’ responses that are related to the direction of the perspective shift. However, it should be noted that the current experiment was not designed to distinguish between egocentric and allocentric reference encoding.

We also demonstrated that the size of the reversed congruency effect can be substantially reduced by adding objects into the environment, such that performance becomes similar across congruent and incongruent trials. In Experiment 3, we tested young and older participants with an environment containing additional objects. Our results showed that older adults were more likely to display the reversed congruency effect. Finally, Experiments 2 and 3 were online studies and provided conceptual replications of the laboratory findings from Experiment 1, showing that online spatial memory and perspective-taking studies using static stimuli can render reliable results.

In Experiment 2, we have shown that the reversed congruency effect was driven by how the object and the camera moved in the environment. Our main explanation for the reversed congruency effect is that camera movements between encoding and test create an induced object motion effect in the same direction as the camera movement. This camera-induced object motion amplifies perceived object movements in incongruent trials and contributes to incorrect responses at smaller object displacement distances in the congruent trials. While it is currently unclear why the movements of the camera lead to the perception of object movement in the same direction, we have shown that the reversed congruency effect is reduced if we provide additional spatial cues by adding further objects to the environment. Increasing the number of cues/landmarks in the environment is associated with improvements in the precision with which object positions are encoded and better understanding of the perspective shifts (Cánovas et al., 2011; Chamizo et al., 2011; Ekstrom & Yonelinas, 2020; Kamil & Cheng, 2001; Luo et al., 2007). As a result, we argue that the camera-induced object motion that gives rise to the reversed congruency effect is driven by difficulties in forming precise representation of object locations and difficulties with understanding perspective shifts can result in camera-induced object motion.

It is possible that the experimental setup that we used, and, in particular, the presentation of a three-dimensional virtual world using two-dimensional images (pictorial space), contributes to difficulties in understanding the position of the object in the environment across different viewpoints. As recently pointed out in other studies (Karimpur et al., 2020; Troje, 2019), the location of the observer in pictorial space is ill defined, because the observer is not actually present in the depicted space (Avraamides & Kelly, 2008). Observers may adopt the location of the (virtual) camera, which is what we asked our participants to do, but this process is challenging for a number of reasons: First, when viewing images, observers have access to monoscopic, but not stereoscopic, depth cues; second, the sensorimotor contingencies that link movements in the world to changes in the retinal image are different for images and for real-world visual space; finally, when viewing pictures, we generally accept distortions of the geometry of the displayed space (Troje, 2019). Together, these factors are likely to contribute to a less reliable understanding of the exact nature of the perspective shift as well as less reliable estimates of the distances and directions to the objects in the stimuli (Karimpur et al., 2020; Troje, 2019). As noted in discussion of Experiment 2, difficulties in depth perception can prevent participants from formulating a correct representation of object location and from understanding the perspective shifts correctly, which in turn may give rise to the camera-induced object motion. Future research should therefore address whether and how the results from our study using pictorial stimuli generalizes to real-world settings or to a setting that makes use of immersive virtual reality.

In Experiment 3, we show that the Reversed Congruency Effect is more pronounced in older adults. Thus, even in situations when additional environment cues are available, older adults have greater difficulties in overcoming the camera-induced object motion. This is in line with our interpretation that susceptibility to camera-induced object motion is driven by difficulties in forming precise representations of object locations and in spatial perspective taking. Specifically, aging is associated with declines in the precision of memory for object locations (Nilakantan et al., 2018; Pertzov et al., 2015; Segen et al., 2020a) as well as difficulties in perspective taking (Montefinese et al., 2015; Segen et al., 2020a). These declines may be associated with age-related functional and morphological changes in the retrosplenial cortex and the hippocampal circuit (Antonova et al., 2009; Langnes et al., 2019; Meulenbroek et al., 2004; Moffat et al., 2007), which is crucial for the development of precise spatial memories (Evensmoen et al., 2013; Nadel et al., 2013) and the manipulation of spatial memories to carry out perspective taking (King et al., 2002).

Our initial aim was to design and test a task that would allow us to study the precision of spatial memory for object locations. However, given the unexpected reversed congruency effect that was observed, further experimentation with the task is necessary to be confident about its validity to serve as a diagnostic tool on spatial memory precision. Specifically, although we managed to substantially reduce the influence the reversed congruency effect has on performance by enriching the environment with additional spatial cues in Experiment 2, the effect was still present in a similar situation in Experiment 3, particularly in older adults. In addition, in Experiment 3 we also showed that individual differences greatly influence performance on our task. It is therefore important that the task is validated using a substantially larger sample and to relate performance on the tasks to spatial cognition tasks. This will allow us to achieve a better understanding of what abilities our task taps into, and to understand what individual differences in spatial abilities are contributing to the observed reversed congruency effect.

Lastly, we should briefly discuss the differences in performance across the three experiments presented here. First, although in Experiment 2 we replicated the congruency effect in the no-additional-objects condition, which most closely resembles Experiment 1, the difference between incongruent and congruent trials was smaller compared with that of Experiment 1. Secondly, performance in the additional-objects condition was lower in Experiment 2 than in Experiment 3. We believe that the differences in performance and the manifestation of the reversed congruency effect between Experiment 2 and Experiments 1 and 3 arise due to the camera always fixating on the target object in Experiment 2. This means that the amount of camera rotation between encoding and test depends on the position of the target object during encoding and the amount of object displacement. In other words, the position of the environmental features in the 2D image differed between trials, even if the camera was in the same position. In Experiments 1 and 3, on the other hand, the camera fixated on the same environmental location, and the positions of all of the environmental features in the image (apart from the target object) are always the same for the given camera positions. These differences in camera rotation may have introduced greater variance in participants’ performance in Experiment 2 and led to overall lower performance compared with Experiments 1 and 3. Despite those fluctuations in performance and in the “size” of the reversed congruency effect, our conjecture is that it is a robust effect that is present across different viewing conditions (e.g., different sizes of monitors used by participants in the online experiments, and relative positions to the monitor) as well as camera settings that are used to render experimental stimuli.

To conclude, in the present study we introduced a novel task to study the precision of spatial memory for object locations across perspective shifts and reported a novel systematic bias in participants’ performance, the reversed congruency effect that is likely to be driven by induced object motion that is introduced by camera movements in the “real world.” We believe that this camera-induced object motion arises from difficulties in formulating precise spatial representations and understanding of perspective shifts. This is in line with our findings across all three experiments showing that the reversed congruency effect is influenced by both environmental properties (i.e., presence of additional cues) and individual differences (age-related differences) that make it harder to understand the spatial perspective shift and the precise position of the objects in the environment. Importantly, our findings highlight that experimental paradigms employing static stimuli across different perspectives can be greatly affected by systematic biases. This has significant implications for the interpretations that can be made from such studies. Thus, researchers should be mindful that camera movements may introduce unwanted systematic biases and given our results, we suggest using environments that contain enough spatial information to enable the formation of precise spatial representations and understanding of the perspective shifts.

## Supplementary Information

ESM 1 (DOCX 741 kb)

## Footnotes

1 Exploratory analyses on sex differences and discussion of results are presented in the Supplementary Material on OSF repository (https://osf.io/7n5vd/).
