# Supplementary material for: Perspective taking and systematic biases in object location memory
Source: Atten Percept Psychophys. 2021 Mar 15;83(5):2033–51. doi: 10.3758/s13414-021-02243-y (PMC7959304; doi:10.3758/s13414-021-02243-y)
Supplement: Supplementary file 1 — (DOCX 741 kb) [file 13414_2021_2243_MOESM1_ESM.docx]

**Supplemental materials**

**Experiment 1 Additional information on 2D strategies**

Screen-based strategy description

The screen-based strategy involves participants memorising the exact pixel position of the target object on the screen and comparing it to the screen position of the object at test (Figure 1A). If participants have relied on this strategy, they would respond correctly on all trials (Figure 1B). However, in situations when the target object moves by small distances, participants would need to be very precise in encoding the position of the target object on the screen as the smallest change in the position of the target object on the screen between encoding and test is 0.49 cm on the horizontal axis of the screen with the widths of 70.5 cm.  As a result, it is possible that in such cases participants would not be able to reliably detect the direction in which the target object has moved on the screen. This does not explain the Congruency effect as even when the target object moves by a similar distance on the screen, performance is still higher for *Incongruent* compared to *Congruent* trials, particularly at smaller distances (Figure 2).

Corner-Based Strategy

In this strategy participants memorise the 2D flattened relationship between the target object and the corner at encoding, and then comparing the same relationship at test i.e. the target object was to the left of the corner at encoding and now appears to be to the right of the corner (in the 2D plain) at test (Figure 1A). If participants solely rely on this strategy, we would expect “maximal” Congruency Effect with ceiling level performance in the *Incongruent* trials and consistent incorrect responses on all (or most depending on which cue participants use i.e. if use poster would make correct responses at 61cm) *Congruent* trials Figure 1B).


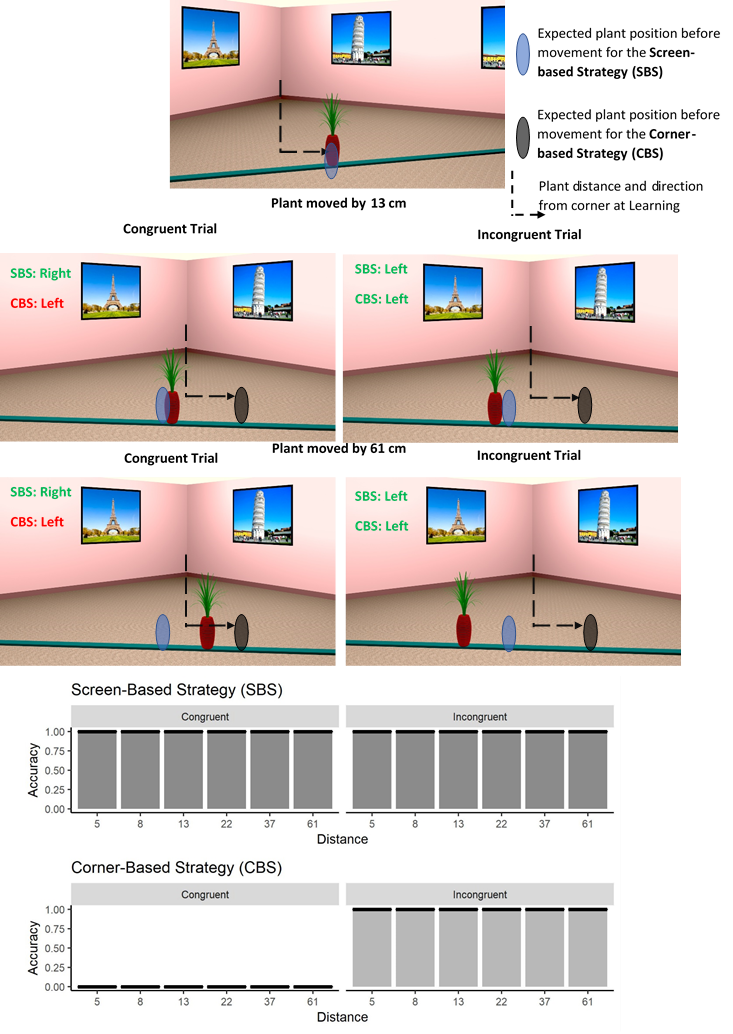


**A**

**B**

Figure 1 A Schematic representing the Screen-Based Strategy (SBS) and the Corner-based Strategy (CBS); B Predicting performance for the SBS (upper panel) and CBS (lower panel) on Congruent and Incongruent trials.


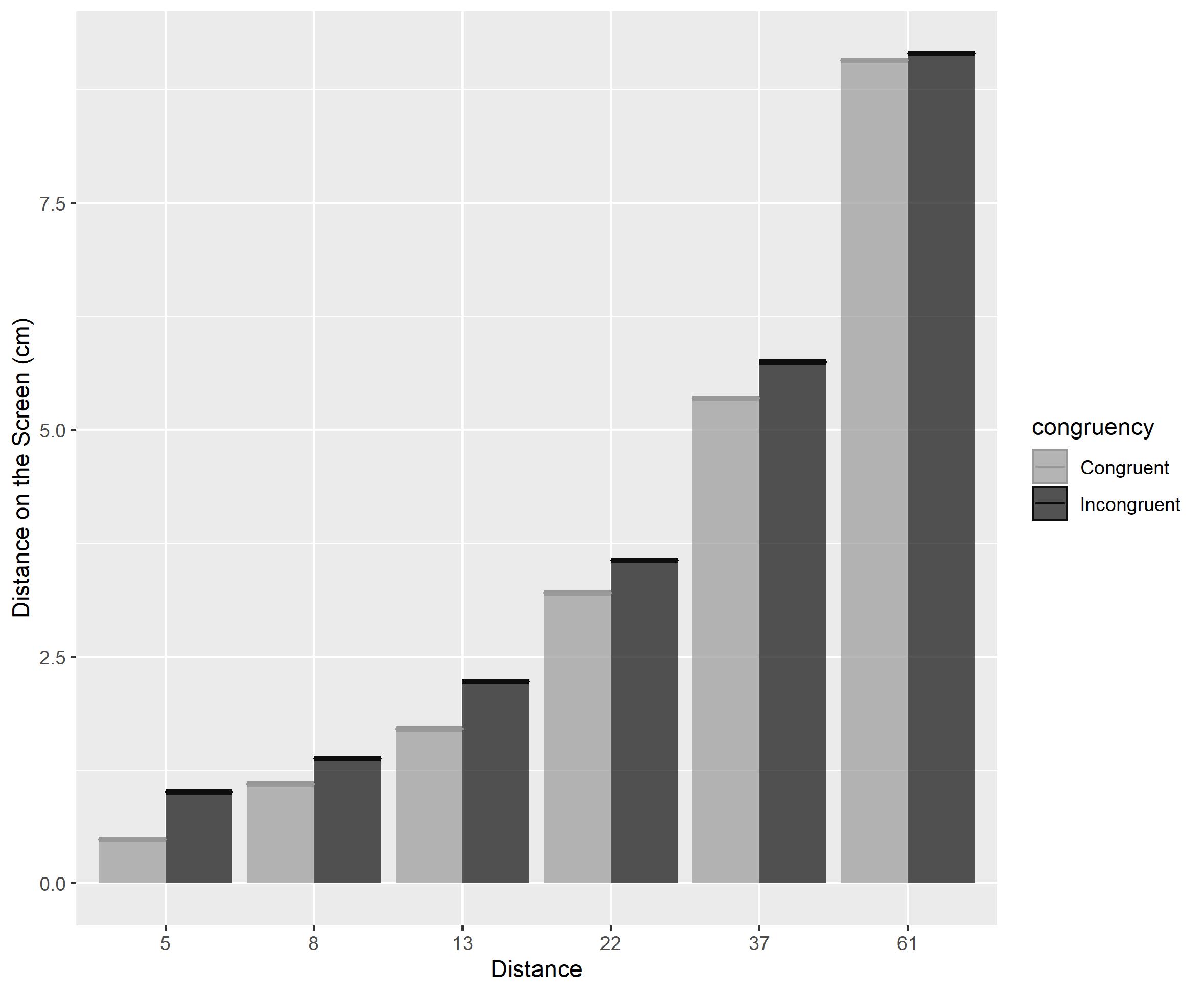


Figure 2 Movement of the object on the screen for Congruent and Incongruent trials as a function of object displacement distance.

### **Follow up analysis of the Congruency Effect in Experiment 2**

To assess the extent to which the addition of objects helped to reduce the Congruency Effect, we split up the data to look at the *No Object* and *Additional Objects* conditions separately. A GLME analysis with Object displacement distance (ODD) and Congruency as fixed effects and a by-item and by-subject intercept and slope for Congruency showed main effects of ODD and Congruency in the *No Object* condition but only a main effect of ODD was found in the *Additional Object* condition. This analysis confirms that the addition of objects eliminated the congruency effect.

Table 1 GLME Coefficient separately for No Objects and Additional Object conditions

|  | Accuracy | | |
| --- | --- | --- | --- |
| Predictors | Coefficients | std. Error | z-value |
| **No Objects** |  |  |  |
| *(Intercept)* | 0.281 | 0.066 | 4.280 |
| log(ODD) | 0.251 | 0.052 | 4.858 |
| Congruency (*Congruent*) | -1.019 | 0.244 | -4.176 |
| log(ODD) * Congruency (*Congruent*) | 0.026 | 0.052 | 0.496 |
| **Additional Objects** |  |  |  |
| *(Intercept)* | 0.784 | 0.095 | 8.240 |
| log(ODD) | 0.521 | 0.062 | 8.344 |
| Congruency (*Congruent*) | -0.345 | 0.299 | -1.152 |
| log(ODD) * Congruency (*Congruent*) | 0.110 | 0.062 | 1.767 |

### **Experiment 2 GLME model with ODD, Congruency, Condition, Rotation and Block as fixed effects**

Table 2 Coefficients from Accuracy GLME analysis

| **Accuracy** | | |  |
| --- | --- | --- | --- |
| *Predictors* | *Odds Ratios* | *std. Error* | *z-value* |
| (Intercept) | 1.397 | 0.039 | 8.665 |
| Log(ODD) | 1.295 | 0.025 | **10**.**317** |
| Congruency (*Congruent*) | 0.667 | 0.025 | **-16.207** |
| Condition (*Additional* *Objects*) | 0.872 | 0.029 | -4.687 |
| Block (*Additional* *Objects 1st*) | 1.055 | 0.038 | 1.396 |
| Rotation (*High*) | 1.028 | 0.025 | 1.116 |
| Log(ODD)* Congruency (*Congruent*) | 1.038 | 0.025 | 1.497 |
| Log(ODD)* Condition (*Additional* *Objects*) | 0.929 | 0.025 | -2.929 |
| Congruency (*Congruent*) * Condition (*Additional* *Objects*) | 0.788 | 0.025 | -9.558 |
| Log(ODD) * Block (*Additional* *Objects 1st*) | 1.031 | 0.025 | 1.243 |
| Congruency (*Congruent*) * Block (*Additional* *Objects 1st*) | 0.947 | 0.025 | **-2.213** |
| Condition (*Additional Objects*) * Block (*Additional Objects 1st*) | 0.945 | 0.029 | -1.946 |
| Log(ODD) * Rotation (*High*) | 1.013 | 0.025 | 0.522 |
| Congruency (*Congruent*) * Rotation (*High*) | 0.852 | 0.025 | **-6.443** |
| Condition (*Additional Objects*) * Rotation (*High*) | 1.047 | 0.025 | 1.825 |
| Block (*Additional Objects 1st*) * Rotation (*High*) | 1.003 | 0.025 | 0.139 |
| Log(ODD) * Congruency (*Congruent*)  * Condition (*Additional* *Objects*) | 0.978 | 0.025 | -0.882 |
| Log(ODD)* Congruency (*Congruent*)  * Block (*Additional* *Objects 1st*) | 1.010 | 0.025 | 0.390 |
| Log(ODD) * Condition (*Additional* *Objects*)  * Block (*Additional* *Objects 1st*) | 0.992 | 0.025 | -0.327 |
| Congruency (*Congruent*) * Condition (*Additional* *Objects*)  * Block (*Additional* *Objects 1st*) | 1.051 | 0.025 | **2.034** |
| Log(ODD)* Congruency (*Congruent*) * Rotation (*High*) | 1.000 | 0.025 | -0.003 |
| Log(ODD)* Condition (*Additional* *Objects*) * Rotation (*High*) | 0.998 | 0.025 | -0.095 |
| Congruency (*Congruent*) * Condition (*Additional* *Objects*)  * Rotation (*High*) | 1.048 | 0.025 | 1.869 |
| Log(ODD) * Block (*Additional* *Objects 1st*) * Rotation (*High*) | 1.023 | 0.025 | 0.906 |
| Congruency (*Congruent*) * Block (*Additional* *Objects 1st*) *Rotation (*High*) | 0.962 | 0.025 | -1.559 |
| Condition (*Additional Objects*) * Block (*Additional Objects 1st*) * Rotation (*High*) | 0.988 | 0.025 | -0.489 |
| Log(ODD) * Congruency (*Congruent*) * Condition (*Additional Objects*) * Block (*Additional Objects 1st*) | 0.976 | 0.025 | -0.991 |
| Log(ODD)* Congruency (*Congruent*) * Condition (*Additional Objects*) * Rotation (*High*) | 1.007 | 0.025 | 0.265 |
| Log(ODD) * Congruency (*Congruent*)  * Block (*Additional Objects 1st*) * Rotation (*High*) | 1.008 | 0.025 | 0.303 |
| Log(ODD)* Condition (*Additional Objects*)  * Block (*Additional Objects 1st*) * Rotation (*High*) | 1.010 | 0.025 | 0.384 |
| Congruency (*Congruent*) * Condition (*Additional Objects*)  * Block (*Additional Objects 1st*) * Rotation (*High*) | 1.036 | 0.025 | 1.434 |
| Log(ODD) * Congruency (*Congruent*) * Condition (*Additional Objects*) * Block (*Additional Objects 1st*) * Rotation (*High*) | 0.973 | 0.025 | -1.086 |

### **Follow-up analysis Experiment 3 Looking at Younger and Older data separately**

We have separated younger and older adult’s data to investigate the nature of the interaction between Age Group and Congruency as well as the interaction between Distance, Age Group and Congruency. To do so we ran two separate GLME models, one for each age group. The key differences between younger and older adults were that older adults showed a greater congruency effect, particularly at smaller distances (Table 3). This suggests that older adults may be more reliant on using 2D strategies at smaller distances when the task demands are higher and greater spatial precision is required.

Table 3 GLME coefficients for younger and older adults

|  | **Accuracy** | | |
| --- | --- | --- | --- |
| *Predictors* | *Estimates* | *std. Error* | *z-value* |
| *Younger Adults* |  |  |  |
| (Intercept) | 2.023 | 0.130 | **15.598** |
| Distance | 1.071 | 0.064 | **16.788** |
| Congruency (*Incongruent*-*Congruent*) | -0.178 | 0.062 | **-2.877** |
| Distance* Congruency (*Incongruent*-*Congruent*) | 0.084 | 0.063 | 1.275 |
| *Older Adults* |  |  |  |
| (Intercept) | 1.474 | 0.152 | **9.711** |
| Distance | 0.852 | 0.050 | **16.972** |
| Congruency (*Incongruent*-*Congruent*) | -0.611 | 0.049 | **-12.575** |
| Distance* Congruency (*Incongruent*-*Congruent*) | 0.365 | 0.049 | **7.382** |
